# First Insights into Human Fingertip Regeneration by Echo-Doppler Imaging and Wound Microenvironment Assessment

**DOI:** 10.3390/ijms18051054

**Published:** 2017-05-13

**Authors:** Paris Jafari, Camillo Muller, Anthony Grognuz, Lee Ann Applegate, Wassim Raffoul, Pietro G. di Summa, Sébastien Durand

**Affiliations:** Plastic and Hand Surgery Department, Lausanne University Hospital, 1011 Lausanne, Switzerland; Paris.Jafari@chuv.ch (P.J.); Camillo.Muller@chuv.ch (C.M.); Anthony.Grognuz@chuv.ch (A.G.); Lee.Laurent-Applegate@chuv.ch (L.A.A.); Wassim.Raffoul@chuv.ch (W.R.); Pietro.Di-Summa@chuv.ch (P.G.d.S.)

**Keywords:** fingertip regeneration, clinical assessment, Doppler imaging, angiogenesis

## Abstract

Fingertip response to trauma represents a fascinating example of tissue regeneration. Regeneration derives from proliferative mesenchymal cells (blastema) that subsequently differentiate into soft and skeletal tissues. Clinically, conservative treatment of the amputated fingertip under occlusive dressing can shift the response to tissue loss from a wound repair process towards regeneration. When analyzing by Immunoassay the wound exudate from occlusive dressings, the concentrations of brain-derived neurotrophic factor (BDNF) and leukemia inhibitory factor (LIF) were higher in fingertip exudates than in burn wounds (used as controls for wound repair versus regeneration). Vascular endothelial growth factor A (VEGF-A) and platelet-derived growth factor (PDGF) were highly expressed in both samples in comparable levels. In our study, pro-inflammatory cytokines were relatively higher expressed in regenerative fingertips than in the burn wound exudates while chemokines were present in lower levels. Functional, vascular and mechanical properties of the regenerated fingertips were analyzed three months after trauma and the data were compared to the corresponding fingertip on the collateral uninjured side. While sensory recovery and morphology (pulp thickness and texture) were similar to uninjured sides, mechanical parameters (elasticity, vascularization) were increased in the regenerated fingertips. Further studies should be done to clarify the importance of inflammatory cells, immunity and growth factors in determining the outcome of the regenerative process and its influence on the clinical outcome.

## 1. Introduction

Humans maintain regenerative capability of fingertips [1,2], replacing the lost tissue following substantial trauma. This regeneration occurs in a level dependent manner as long as the proximal nail matrix remains intact [3]. Regenerative mechanisms in humans are not elucidated but the involvement of Wnt activation in nail stem cells and fibroblast growth factor 2 (FGF-2) signaling have been described in mice and amphibian models for limb regeneration [3,4,5,6]. Fingertip regeneration derives from proliferative mesenchymal cells (blastema) [7] that subsequently differentiate into soft and skeletal tissues [8,9]. In humans the conservative treatment of the amputated fingertip under occlusive dressing is essential to shift the response of tissue loss from a wound repair process towards regeneration [10]. Therefore, the regenerative microenvironment created under the occlusive dressing should play an important role in providing signals that initiate regeneration and control proliferation and patterning [11]. Following skin injury in humans, immediate release of various growth factors, cytokines and chemokines triggers and controls sequential steps of wound repair. The role and importance of these factors in the repairing microenvironment of wounds has been well established [12]. Among these growth factors, platelet-derived growth factor (PDGF) is known to have a central role in all different steps of wound repair by triggering cell migration into the wound, enhancing the proliferation of fibroblasts and extracellular matrix production and also contraction [13]. Epidermal growth factor (EGF) family of growth factors is also important for reepithelialization and vascularization during wound repair [12,14]. Vascular endothelial growth factor (VEGF) and other growth factors that increase expression such as hepatocyte growth factor (HGF) [15] have crucial roles in wound angiogenesis [16] which then affects overall wound repair processes [17]. Apart from angiogenesis, nerve in-growth is also important in wound healing [18] and the growth factors that have an effect on innervation such as nerve growth factor (NGF) have been shown to be essential to normal wound repair [19]. Along with several growth factors, chemokines such as macrophage inflammatory protein 1-α (MIP 1-α) play an important role in the recruitment of inflammatory cells to the wound site [20] and initiation of wound repair processes [21] within the inflammatory phase that implicates several proinflammatory cytokines such as IL1-α, IL1-β, IL-6 and TNF-α [22]. There might be similarities between the implication of the above mentioned factors between wound repair and wound regeneration. However, knowledge on the role of these factors during regeneration is lacking and limited to animal models. PDGF is shown to promote the expansion of the blastema mesenchymal precursor population which is differentiated to bone and dermis [23] while VEGF is considered to be inhibitory for regeneration [24]. The implication of immune signaling in regeneration is shown in axolotl model of regeneration with an increase in the expression of several proinflammatory cytokines early after limb amputation [25]. However, cellular and molecular mechanisms in human fingertip regeneration remain poorly understood. In this preliminary study, we investigated the early pro-regenerative microenvironment of human fingertip amputations treated with occlusive dressing. Our primary purpose was to determine factors that favor regeneration over repair in these wounds. In three consecutive patients, growth factor, chemokine and cytokine contents of the regenerating wound fluid were analyzed. Since burn wounds are considered to be repairing rather than regenerating wounds [26], we compared the fingertip wound fluid cytokine and growth factor content, with burn wound fluid from three burn patients. Our secondary goal was to assess the quality of the regenerated tissue. We quantified the functional, vascular and mechanical properties of the regenerated fingertips three months after trauma and compared the data to the corresponding fingertip on the collateral uninjured side. Clinical (sensory recovery), morphological (pulp thickness and texture) and mechanical parameters (elasticity, vascularization) were recorded and analyzed.

## 2. Results

### 2.1. Clinical Evaluation of Regenerated Fingertips

**Figure 1:**
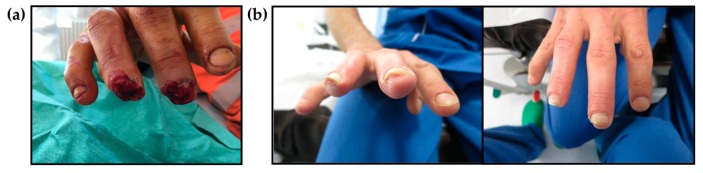
Representative images of amputated fingers. (**a**) At admission and before the application of occlusive dressing; (**b**) Three months post-trauma and at clinical and morphological evaluation.

Five healthy male patients (mean age 50 ± 15 years) were included in this preliminary study (Table 1). The occlusive dressing was applied upon admission (Figure 1a) and after primary debridement and changed once a week without rinsing the wound for a mean duration of 5 weeks (4–7). Patients had 9 (7–12) consultations within the first 6 months. Mean follow-up was 6 months (5–7). Clinical and morphologic evaluation was done at three months post-trauma (Figure 1b).

Sensitivity of the regenerated fingertips was assessed by two points discrimination that was measured less than 4 mm and overlapped with the uninjured side for each finger. We assessed eventual pain using the Verbal Descriptor Scale representing different growing intensities of pain varying from none to very severe pain. Our patients had no or mild pain in the regenerated fingertips. Thumb-finger Pinch was recorded as an index of functional force that did not reveal substantial difference with collateral control fingers.

### 2.2. Evaluation of Morphological, Mechanical and Vascular Properties of Regenerated Fingertips

We used different modalities of Ultrasound imaging (aixplorer^®^, Aix-en-Provence, France) to measure the above parameters. Pulp thickness assessment was based on B-mode echography, pulp vascularization was measured by Doppler ultrasound test and the elasticity of the regenerative tissue was assessed by the elasticity modulus from Shear Wave Elastography (SWE).

**Figure 2:**
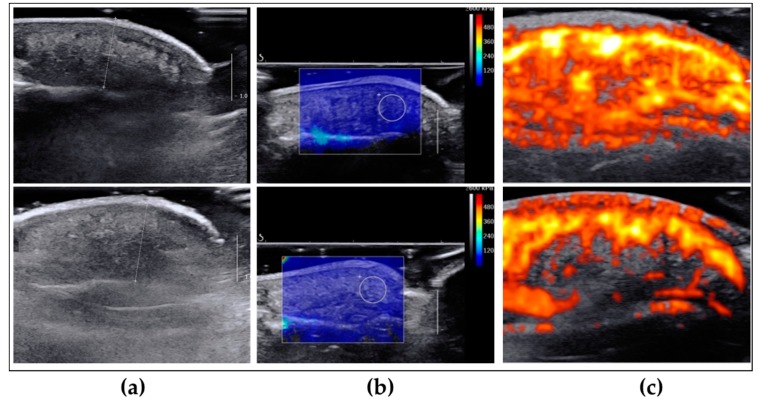
Representative echo-doppler images of regenerated (upper row) and un-injured collateral (lower row) fingertips. (**a**) Ultrasound b-mode imaging for the measurement of the pulp thickness; (**b**) b-mode imaging with superimposed share wave mapping for the measurement of the pulp elasticity. The white circle has a diameter of 5 mm; (**c**) b-mode imaging with superimposed power Doppler for the measurement of vascularity.

**Figure 3:**
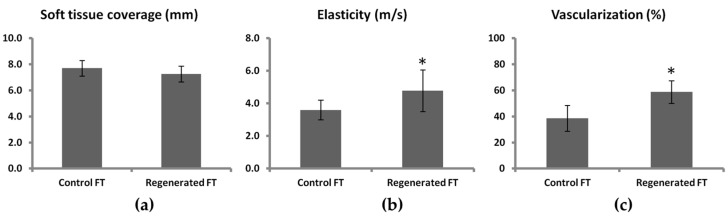
Morphologic, mechanical and vascular characteristics of regenerated fingertips compared to control collateral healthy finger. (**a**) Soft tissue coverage, (**b**) Elasticity, (**c**) vascularization. Data are presented as mean ± SD for five patients (* *p* < 0.05).

We observed no significant differences in pulp thickness between regenerated fingertips and the collateral control finger (Figure 2a and Figure 3). The average soft tissue coverage was 7.2 ± 0.6 mm (6.7–8.2) compared to 7.7 ± 0.6 mm (6.9–8.3) in control collateral finger with no significant difference observed. Interestingly, elasticity (Figure 2b and Figure 3) was measured 4.8 ± 1.3 m/s (3.5–6.7) compared to 3.6 ± 0.6 m/s (3.1–4.4) in control collateral fingers and this represented a significant increase of 132.3 ± 23% in regenerated fingertips. Likewise, vascularization (Figure 2c and Figure 3) was significantly higher (162.4 ± 53.8%) in the regenerated fingertip 58.8 ± 8.6 % (50.7–72.1) compared to controls with a vascularization percentage of 38.6 ± 10% (26.3–51.4).

### 2.3. Regenerative Wound Microenvironment

**Figure 4:**
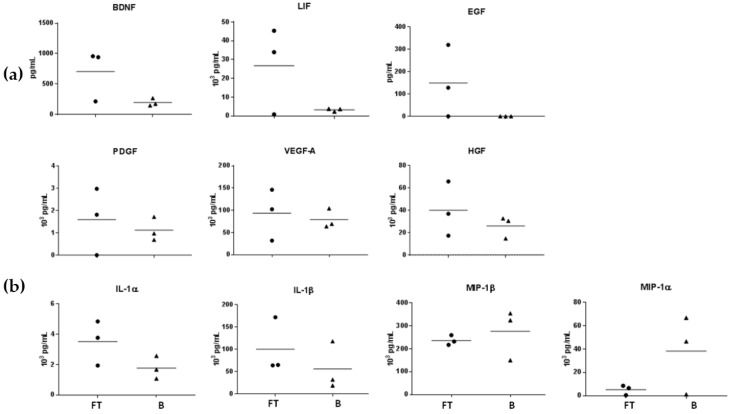
Measured levels of (**a**) growth factors, (**b**) cytokines and chemokines in fingertip exudate samples (FT) and burn wound exudate samples (B), seven days post-trauma.

We measured the concentrations of different inflammatory cytokines, chemokines and growth factors in the wound exudate from three amputated fingertips and three burn patients using Luminex technology. Despite a large variation in wound cytokine levels among different patients (Figure 4), the concentrations of brain-derived neurotrophic factor (BDNF), EGF and leukemia inhibitory factor (LIF) were higher in fingertip exudates than in burn wounds. VEGF-A, PDGF and HGF were highly expressed in both samples in comparable levels (Figure 4a). We observed a general trend towards higher values of inflammatory cytokines in fingertip exudates whereas chemokine levels of burn wound exudates were globally higher (Figure 4b). Bone Morphogenetic Protein signaling has been shown to be important during fingertip regeneration in mice [27] but we did not observe detectable levels of BMP in the fingertip exudate samples.

## 3. Discussion

General belief is that the ability to regenerate fingertips is lost or decreased in human by age [10]. Here we show that in all our adult patients (mean age 50 years) fingertips underwent regeneration with satisfactory clinical outcome. By definition, re-growth of cells or tissues during regeneration replace both form and function in damaged organs [28]. Indeed, clinical assessment of the regenerated fingertips in our patients revealed that they have comparable morphological and functional characteristics with contralateral uninjured fingertips. However, we observed key mechanical modifications in the regenerated fingertips. Increase in elasticity paralleled high vascularization in regenerated fingertips suggesting that the enhanced vascularity during regeneration affects the elasticity of these tissues. Nevertheless, long-term studies are necessary to confirm whether these events are associated and if they persist in time after the regeneration process is completed. Increased vascularization could be transient and a result of increased expression of pro-angiogenic factors in the regenerative microenvironment of the fingertips as we observed in this study. We analyzed the wound fluid that was accumulated under the occlusive dressing over amputated fingertips over the primary phase of regeneration and most probably blastema formation [29]. We measured high levels of VEGF in the wound fluid that explains increased vascularity which seems to be essential for the complete regeneration in human fingertips. Interestingly, in mouse models of regeneration, an inverse phenomena is observed where the expression of VEGF is down regulated in blastema [11] and even induction of angiogenesis by VEGF inhibited the regeneration by altering the transition of blastema to the differentiation phase [24]. Thus, for the first time, our results reveal discrepancies between mice and human fingertip regeneration. However, in a recent study, regenerated fingertips in fast-healing mice showed 70% increased vascularization compared to non-healing mice with impaired regeneration [30]. These data along with our observations, suggest an important role for higher blood flow in the fingertip regeneration in mammalians. In surgical wounds and chronic vascular ulcers, high expression of b-FGF, which is another pro-angiogenic factor, is observed at very early phases of wound healing and the levels further decline over-time during healing [31,32]. We did not detect this growth factor in the accumulative exudates collected at day 7, which is most probably too late for b-FGF expression. A more comprehensive analysis of pro-angiogenic factors over time in fingertip exudate is necessary and will help to determine the factors that control angiogenesis during regeneration.

Deficiency in inflammatory response has been suggested to be an explanation for the absence of fibrotic response during fingertip regeneration [33]. However, our preliminary data showed that, at least during the first seven days post-amputation in human fingertips, a strong inflammatory response is ongoing in the regenerative microenvironment compared to healing microenvironment of burn wounds as shown in Figure 4. The central role of inflammation and innate immune response are known in wound healing but still poorly understood in regeneration models [34], where a prominent expression of inflammatory and immune-related genes was revealed [35,36]. In these models, both pro- and anti-inflammatory cytokines are highly expressed during the first two weeks of regeneration with pro-inflammatory molecules being prominent during the first week and anti-inflammatory cytokines at the second week and after [34]. Classical models of regeneration such as salamander [25], Xenopus [35] or mice digit tip [37] also show that protein factors triggering the immediate events following traumatic injury are similar in both wound healing and organ regeneration [25,35], but the immune factors that favor one pathway over the other remain unknown. In our study, pro-inflammatory cytokines were relatively higher expressed in regenerative fingertips than in the burn wound exudates while chemokines where present in lower levels. Further studies should be done to clarify the importance of inflammatory cells and immunity in determining the outcome of the regenerative process.

In amphibians and mice, complete regeneration is dependent to nerve-derived growth factors that are secreted by nerve-associated Schwann cell precursors and promote the expansion of blastema and digit regeneration [38,39]. Transcriptome analysis these cells revealed that PDGF, LIF and could be potential paracrine factors that might be important for digit tip regeneration [23]. We observed an increased expression of LIF and BDNF in the regenerating fingertip exudates compared to burn wound exudates. The role of enervation as a regeneration triggering factor in humans is not known but our clinical observations with restoration of normal sensitivity seems to reveal an important nerve regeneration process (supported by increased expression of growth factors such as BDNF) to be active in human fingertips. Application of occlusive dressing leads to the creation of local permissive niche that provides necessary signals to initiate regeneration which is influenced by local microenvironment rather than systemic factors [40].

We detected the expression of other growth factors that promote wound reepithelialization, in fingertip exudates such as HGF and EGF that should have a role in the proliferation and further redifferentiation of blastema cell mass. Further work with higher number of patients is required to establish eventual difference between growth factor profiles and signaling differentiation pathways in regenerating or healing wounds. Moreover, in this study we were focused only on the first week post-amputation that we considered being crucial for the initiation of regeneration and blastema formation in regenerative fingertips. In further studies, it would be important to perform sample collection at different time points of regeneration in order to investigate which signaling molecules are involved in different phases of regeneration and how their expression might change over time.

## 4. Materials and Methods

### 4.1. Ethics

Written consent was obtained from all patients, and the procedures were performed in line with the Helsinki Declaration of 1975 (Recommendations guiding medical doctors in biomedical research involving human subjects). Exudate collection were approved by the State Ethics Committee (Protocol 488/13) and regulated by the DAL (Department of Musculoskeletal Medicine) and Institutional Biobank Procedures.

### 4.2. Fingertip Exudate Collection and Analysis

Fingertip exudates were collected from occlusive dressing at the first dressing change seven days after trauma. Samples were diluted in assay buffer, centrifuged and the supernatants were stored at −80 °C until further analysis.

### 4.3. Burn Wound Exudate Collection

Burn wound exudate was collected as described in [41] and with ethical approval. Briefly, a negative pressure dressing was applied over second-degree burn wounds that collected the accumulated fluid into a reservoir bottle. Samples were collected by changing the reservoir bottle and storedat −80 °C until further analysis.

### 4.4. Wound Exudate Immunoassay

Wound exudate levels of different cytokines, chemokines and growth factors were quantified using Luminex analysis on samples collected from three patients with regenerating fingertips under occlusive dressing and three burn patients according to the instructions of the manufacturer (R&D systems, Minneapolis, MN, USA). Absorbance was read on a Luminex 200 IS device and absorbance data were converted to protein concentrations with Luminex 100 IS Software (version 2.3, Luminex, Oosterhout, The Netherlands). Patient samples were tested in triplicate, and the mean value was used for analysis.

### 4.5. Clinical Assessment of the Regenerated Fingertips (Two Point Discrimination, Force and Pain)

Two-point discrimination was performed with the 2pt Disk-criminator from Alimed^®^. Control finger was tested first and regenerated fingertip afterwards. Fingertips were tested in 1 and 2 point discrimination. The patient, with closed eyes, stated whether he felt one or two points. We began the test at 15 mm distance and then moved the two points closer until the patient could not discriminate anymore (until 4 mm).

Two fingers round pinch (tip to tip pinch) was measured using 50 lb hydraulic pinch gauge (FE-120601) Jamar^®^. The gauge was place between the tip of the thumb and the tip of the concerned finger. The patient applied a spark of maximal power. Pain assessment was performed using visual descriptor scale representing different growing intensities of pain varying from zero to five (0 corresponding to no pain; 1 mild pain; 2 moderate pain; 3 severe pain; 4 very severe pain and 5 worst pain imaginable).

### 4.6. Power-Doppler and Elastography Assessment of Regenerated Fingertips

Digital pulp thickness assessment was based on B-mode echography (aixplorer^®^, Aix-en-Provence, France). Pulp vascularization was measured by power doppler ultrasound test and the elasticity of the regenerative tissue was assessed by the elasticity modulus from Shear Wave Elastography (SWE). Power Doppler sonography is a technique that displays the strength of the Doppler signal in color, rather than the speed and direction information. It has three times the sensitivity of conventional color Doppler for detection of flow and is particularly useful for small capillaries with low-velocity flow. SWE on the other hand is a technique measures the elasticity of tissue by measuring the propagation speed of shear waves, resulting in mechanical disturbances of ultrasonic waves applied to the tissue by the ultrasound probe [42].

The SWE measurements were taken using equipment (Aixplorer^®^, Aix-en-Provence, France), with a high-frequency SHL 15-4 probe (medium frequency 12 kHz). We first used normal ultrasound imaging in B-mode for topographic orientation and then superimposed and further shear wave elastography mapping in penetration mode. A mapping of the shear wave velocity, from blue (soft tissue, low speed) to red (hard tissue, high speed), produced the first qualitative information. A 3 mm^2^ Q-Box (quantitative box) focus area was established in the middle of the digital pulp, and the quantitative values were obtained in m/s (shear wave velocity) and in kPa (elasticity modulus). To asses microvascularisation we performed echo-doppler in the same manner. After positioning of the ultrasound probe in B-mode, and then superimposed the ultrasound Doppler mapping. All images were saved in dicom format.

### 4.7. Image and Statistical Analysis

The pulp vascularization of regenerated fingertips and of collateral control fingers was observed by Doppler ultrasound (aixplorer^®^, Aix-en-Provence, France) and images were recorded as DICOM files. In absence of vascularization, the pixels remained gray or black. Regions that were vascularized appeared in color varying from red to white depending on the intensity. Images were processed within ImageJ software (NIH, Bethesda, MD, USA) to extract vascularization pixels which were selected with a color threshold based on Hue Saturation and Brightness (HSB) color mode. Pixels with Hue between 0 and 60, saturation between 5 and 255 and brightness between 15 and 255 were selected and considered as vascularization. The percentage of vascularization was calculated as a ratio between vascularization pixels divided by total number of pixels in the evaluation zone.

Independent 2-sided *t* test was used to evaluate statistical difference between the morphological and mechanical characteristics of regenerated fingertips and control healthy fingers. *p* < 0.05 was considered as significant.

## Figures and Tables

**Table 1:** Patient and amputated fingertip characteristics.

Patient	Age	Amputated Finger/Hand	Two Points Discrimination	Pinch Test	Pain (0–5)
Patient 1	30	Middle/left	≤4 mm on both fingers	2 kg/2 kg	0/0
Patient 2	45	Middle/right	≤4 mm on both fingers	2.5 kg/2 kg	0/0
Patient 3	76	Index/right	≤4 mm on both fingers	Not obtained	0/0
Patient 4	47	Middle/left	≤4 mm on both fingers	Not obtained	0/0
Patient 5	52	Middle/right	≤4 mm on both fingers	1.5 kg/2.5 kg	1/0

## Abbreviations

FGF-2	Fibroblast Growth Factor 2
SWE	Sear Wave Elastography
BDNF	Brain-derived Neurotrophic factor
PDGF	Platelet-Derived Growth Factor
VEGF-A	Vascular Endothelial Growth Factor A
NGF	Nerve Growth Factor
b-FGF	Basic Fibroblast Growth Factor
MIP	Macrophage Inflammatory Protein
LIF	Leukemia Inhibitory Factor
CNTF	Ciliary Neurotrophic Factor
HGF	Hepatocyte Growth Factor
EGF	Epidermal Growth Factor
